# A Novel Wearable Soft Glove for Hand Rehabilitation and Assistive Grasping

**DOI:** 10.3390/s22166294

**Published:** 2022-08-21

**Authors:** Yinlong Zhu, Weizhuang Gong, Kaimei Chu, Xu Wang, Zhiqiang Hu, Haijun Su

**Affiliations:** 1 College of Mechanical and Electronic Engineering, Nanjing Forestry University, Nanjing 210037, China; 2 State Key Laboratory of Robotics, Shenyang Institute of Automation, Chinese Academy of Sciences, Shenyang 110169, China; 3 Department of Mechanical and Aerospace Engineering, Ohio State University, Columbus, OH 43210, USA

**Keywords:** soft rehabilitation gloves, soft actuator, wearable devices, soft robot

## Abstract

In order to assist patients with finger rehabilitation training and grasping objects, we propose a new type of soft rehabilitation gloves (SRGs), which has both flexion/extension and abduction/adduction movement function for every finger. This paper describes the structure design of the bending actuator and rotating actuator, the fabrication process of the soft actuator, and the implementation of the soft wearable gloves based on a fabric glove. FEM simulation analysis and experiments were conducted to characterize the mechanical behavior and performance of the soft glove in terms of the angle output and force output upon pressurization. To operate this soft wearable glove, we designed the hardware system for SRGs with a flexible strain sensor and force sensor in the loop and introduced a force/position hybrid PID control algorithm to regulate the pressure inputted. Experiment evaluation focused on rehabilitation training gestures; motions and the precise grasping assistance function were executed. The rotating actuator between each finger can supply abduction/adduction motion manner for patients, which will improve rehabilitation effect. The experimental results demonstrated that the developed SRGs have the potential to improve hand movement freedom and the range of grasping successfully.

## 1. Introduction

In people’s daily living activities, hands play an important role. However, a stroke, incomplete spinal cord injury, brachial plexus injury, Parkinson’s disease, or muscular dystrophy may cause impairment of the hand, which will lead to the loss of hand function [1,2,3]. Car accidents or work injuries may also result in hand impairments [4]. A practical solution for patients would be rehabilitation exercises [5], whereas the lack of rehabilitation devices and one-on-one counseling treatment supplied by medical practitioners usually cannot guarantee the training intensity for patients [6]. Over the past decades, developments in robotics have enabled them to be an important part in the rehabilitation training process [7].

So far, two classes of rehabilitation devices are the focus of research: rigid hand exoskeletons [8,9,10] and soft assistive gloves [11,12,13]. Traditional rigid robots, actuated by electric motor, are commonly characterized by complex mechanism design and high weight, which would introduce uncomfortable experience and hidden danger for patients. Compared with rigid rehabilitation robots, soft wearable rehabilitation gloves are fabricated by soft materials, mainly driven by cable, intelligent material, or a pneumatic/hydraulic elastomeric actuator, which enables a jointless soft structure for the hand to be comfortable and much safer. With many advantages such as their light weight, portable nature, high power-to-weight ratio, low cost, and excellent human–machine interaction, soft wearable gloves are expected to be more suitable and prospective for hand rehabilitation training than their rigid counterparts. Soft rehabilitation gloves can bring certain positive effects and improve the patient’s hand rehabilitation treatment, reduce the economic burden of patients, and enable the patient to return to society and family as soon as possible. Inspired by the bionic method, soft joint structure based on composite fabric material is introduced for the design of the soft glove [14]. 3D printing methods could produce rather light soft gloves capable of flexion and extension [15]. Deep learning could be adopted for position estimation and control of soft gloves [16]. Force feedback gloves were also proposed for generating haptic signals, which could make the remote or virtual working tasks more specific and controllable [17].

Soft robot actuation mainly includes pneumatic/hydraulic drive [18], cable/tendon drive [19], and other smart material drives [20]. As for pneumatic- or hydraulic-actuated soft wearable gloves, polymers or fabric materials are used to fabricate embedded chambers that can generate desired motion manners such as bending and extending upon pressurization. In recent years, several groups have explored various soft gloves for hand rehabilitation training. The most representative of them is the Harvard University group. Polygerinos et al. [21] utilized soft actuators consisting of elastomeric chambers with fiber reinforcements to design a soft wearable glove that generates bending motion, twisting, and extending trajectories under fluid pressurization. Yap of National University of Singapore [22] presented a soft robotic glove based on fabric material for hand-impaired patients for rehabilitation training and assistance with activities of daily living. The glove provides bidirectional motion manners including both active finger flexion and extension with a folded chamber wrapped inside to enlarge the bending angle. Gu group [23] of Shanghai Jiao Tong University developed a high-force fabric-based pneumatic actuator with asymmetric chambers fabricated by two different layers of textiles and introduced interference pads on adjacent-pleat surfaces to improve the stiffness of the pneumatic actuators and heighten the output force. Cappello [24] developed a fabric-based rehabilitation glove by using two fabric layers of unidentical materials. Through varying fabrics and their layered arrangements based on air bags inside, the glove could assist hand opening and closing. In a biomimetic study, Korean D. H. Kim et al. [25] developed a circular finger stretching mechanism for hand rehabilitation, which mimicked the origin, structure, and tendon orientation of the extensor tendon. Ning Guo [26] of the University of Hong Kong designed a rehabilitation glove using a non-deformable rod as the bone and the deformable soft actuator part as a separate part. To further improve the sensing property, PDMS may be considered to be adopted on the skin of the soft hand due to its convenient regulated electrical performance [20].

Despite the above impressive achievements, these soft wearable gloves mainly focus on flexion and extension of five fingers; only the Gu group [23] paid attention to the thumb abduction assistance. However, the abduction motion of the other four fingers is also required to further improve the rehabilitation efficacy and enlarge assistance function. Until now, few works have studied the effects of the abduction motion of all five fingers.

In this paper, we present a novel soft rehabilitation glove that can provide flexion, extension, abduction, and adduction motion for every finger. The whole system comprises a fabric glove, five bending actuators, and four rotating actuators and was fabricated with soft materials. In Section 2, according to the design requirements of hand rehabilitation, the structure design of the two classes of soft actuator were presented, and fabrication of the soft actuator were conducted by using the silicone casting and the lost wax method. In Section 3, finite element analysis for the different structural parameters of the soft actuator on the deformation were conducted, and the simulation results were utilized to optimize the structure of the rotating actuator. Based on the simulation results, the workspace of a single bending actuator and the entire rehabilitation glove were depicted using Matlab software. In Section 4, experiments were performed to test the angle and force output of the bending actuator and rotating actuator; the experimental results agree well with the finite element simulation. In Section 5, the control system hardware and the human–computer interaction interface of the SRGs were designed and the force/position hybrid PID control algorithm was realized. Finally, in Section 6, experiments on rehabilitation training range and precision grasping ability of the SRGs were all conducted. Performance evaluation shows the presented SRGs have good flexibility and adaptability for hand wearing, which can assist patients to fulfil rehabilitation training and provide grasping assistance to improve daily living level. In order to achieve convenient, efficient, and economic rehabilitation training strategies for hands, the existing method still needs to be further improved.

## 2. Structure Design and Fabrication of Soft Gloves

The human hand is a complex and flexible organ in the human body, which has five fingers including the thumb, index finger, middle finger, ring finger, and little finger. The hand has a total of 29 muscles, 19 bones, and 19 joints, with 22 degrees of freedom, which provides excellent operability, perception, and adaptability. The size of each person’s hand is different. Finger movement is limited by physiological structure and has coupled motion with joints. There are two main movement modes of the fingers: flexion/extension and abduction/adduction.

### 2.1. Structure of Soft Gloves

**Figure 1:**
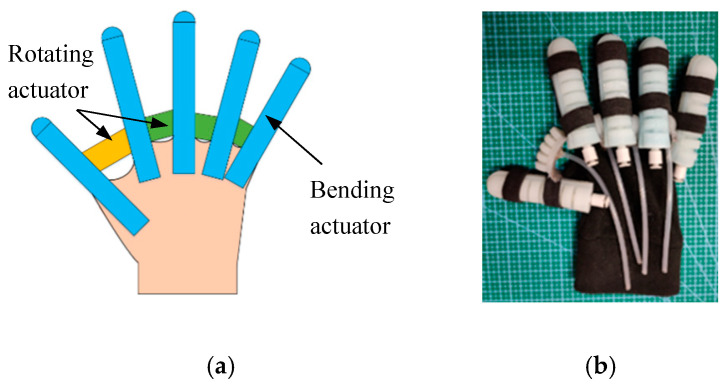
Soft Rehabilitation Gloves. (**a**) Schematic of SRGs and (**b**) Prototype of SRGs.

SRGs are a combination of soft bending actuators and rotating actuators based on fabric gloves to achieve finger rehabilitation training and grasping by controlling the soft actuators. As shown in Figure 1, the SRGs consist of five bending actuators and four rotating actuators. These soft actuators are sewed on a fabric glove with some elastic bands, five bending actuators are fixed on each finger, a big rotating actuator is sewed between the thumb finger and index finger, and three small rotating actuators are stitched between the other four fingers.

**Figure 2:**
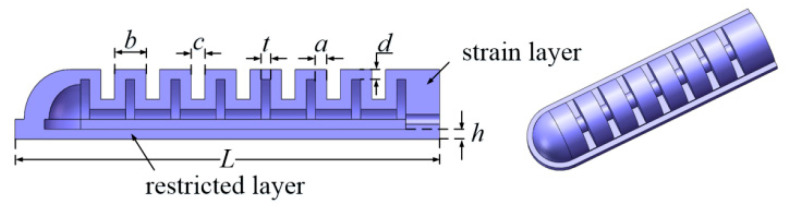
Structure of the bending actuator.

The bending actuator is utilized to realize the flexion and extension motion when inflated or deflated with compressed air. The structure of the bending actuator is shown in Figure 2, which consists of a strain layer with a semicircular cross-section, a restricted layer, and a series of air chambers. When compressed air is filled in the airway, the actuator bends to the restricted layer. The parameter value set for the four-finger bending actuator is listed in Table 1.

**Figure 3:**
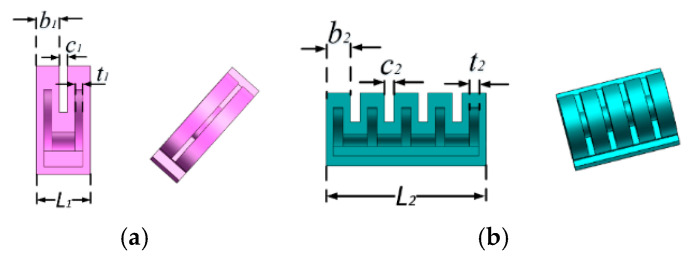
Structure of rotating actuators. (**a**) Rotating actuator for four fingers and (**b**) Actuator for thumb.

The rotating actuators among five fingers have the similar structure as the bending actuator, which are used to adjust the range of angle between each finger. Because the motion range of the thumb is much greater than others, a rotating actuator with more air chambers is fixed between the thumb and the index finger. The rotating actuator has a compact initial shape, and its structure is shown in Figure 3; the actuator for the index finger to the little finger has two chambers and the actuator for the thumb finger has four chambers. The parameter value set for the thumb rotating actuator is listed in Table 2.

### 2.2. Fabrication of Actuators for Soft Gloves

**Figure 4:**
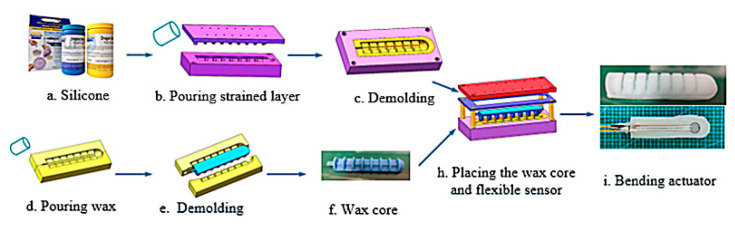
Manufacturing process of the bending actuator.

The manufacturing process of the bending actuator is mainly through the curing and molding of silica gel, combined with the lost wax method. The production process is as follows:

As shown in Figure 4, the A and B parts of silicone (Dragon Skin 20 by Smooth on Inc., Macungie, PA, USA.) were mixed with a ratio of 1:1 and then stirred in a mixer to remove foam. The silicone was then poured into the mold for the strain layer and solidified after 4–5 h. Meanwhile, the melted wax was poured in the mold to fabricate the wax core. Finally, the wax core, the flexible strain sensor, and the force sensor were placed into the molds to cure. Finally, the bending actuator was placed into the oven (95°) for 5 h to melt the wax core.

**Figure 5:**
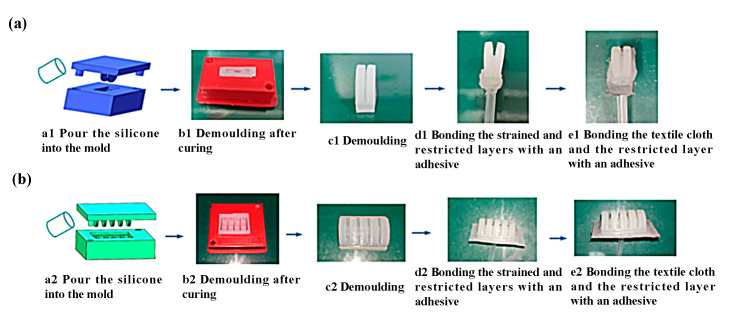
Fabrication process of rotating actuator: (**a**) the normal rotating actuator and (**b**) the thumb.

As can be seen form Figure 5, the fabrication process of rotating actuators is similar to that above for bending actuators. Compared to the fabrication of a bending actuator, there is no step of making a wax mold. For convenience of sewing, the fiber cloth was pasted on the surface of the limiting layer.

## 3. Finite-Element Analysis and Workspace of Soft Gloves

Compared to the mathematical model, the Finite element method can quickly depict the response of an actuator for complex configuration and illustrate nonlinear behavior of the soft actuator such as the interaction of internal layers of different materials.

To guide the design of the soft actuator, finite element simulation needed to be performed on the soft actuator to analyze the influence of the structural parameters of the soft actuator on its performance. For hyperplastic materials such as silicone, it is usually assumed to be incompressible and isotropic, and the phenomenological theory is usually used in the analysis. Here, the Yeoh form strain energy function was used to describe the mechanical behavior of the soft actuator, and it can be shown as

(1) $$W=\sum_{i=1}^{2}C_{i0}{\left(I_{1}-3\right)}^{i}$$

where *I*_1_ is the deformation tensor, dimensionless.

(2) $$I_{1}=\lambda_{1}^{2}+\lambda_{2}^{2}+\lambda_{3}^{2}$$

*λ_i_* (*i* = 1, 2, 3) represents the main elongation ratio in each direction, dimensionless. The true principal stress (also known as the Cauchy stress) is the force per unit area after deformation; *σ_i_* (*i* = 1, 2, 3) can be obtained by taking the partial derivative of the principal elongation ratio through the strain energy function:

(3) $$\sigma_{i}=\lambda_{i}\frac{\partial W}{\partial \lambda_{i}}-p_{0}=2\lambda_{i}^{2}\left[C_{10}+2C_{20}(I_{1}-3)\right]-p_{0}$$

*p*_0_—the hydrostatic pressure to keep the volume constant.

**Figure 6:**
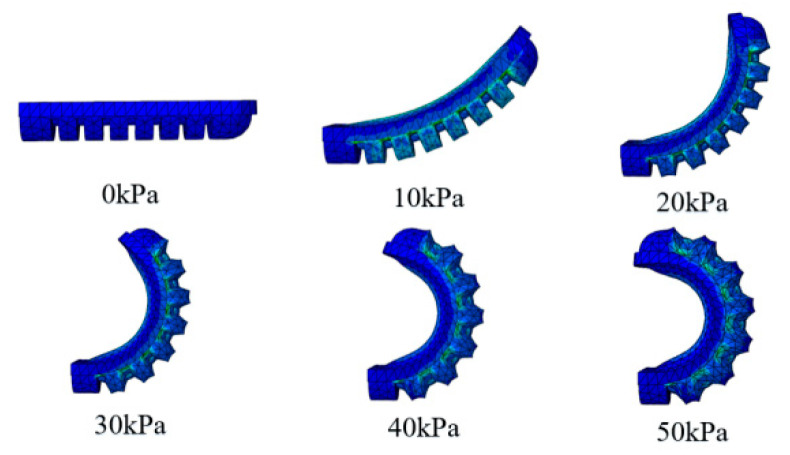
Simulation results of bending actuator.

The material parameters *C*_10_ and *C*_20_ for Dragon skin 20 (*C*_10_ = 0.1 1MPa, *C*_20_ = 0.02 MPa) [27] material was determined by fitting the uniaxial tensile curve. All the components of the actuator were modeled using solid tetrahedral quadratic hybrid elements C3D10H. The ABAQUS simulation results of the bending angle for the soft bending actuator under various pressure increments are shown in Figure 6. It can be seen that the bending angle under 50 kPa is up to 130°, which can meet the rehabilitation requirement.

**Figure 7:**
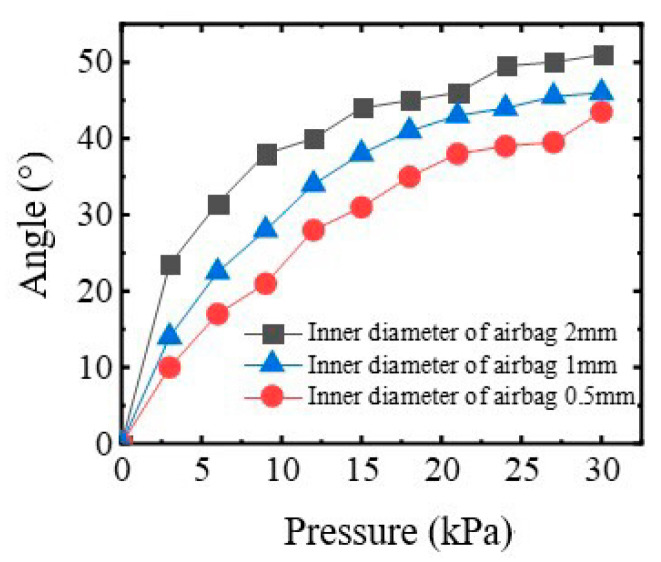
The bending angle under various pressure.

The influence of the inner diameter of the chamber on the angle of bending actuator for four fingers is shown in Figure 7. It can be seen that with the increase of applied air pressure, the bending angle increases nonlinearly and monotonically. Under the same pressure, the larger the inner diameter of the air chamber, the greater the deformation angle. Taking into account the range of angular variations between the four fingers and the safety of the rehabilitation training, the inner diameter of the air chamber of the rotary actuator is taken as 1 mm.

**Figure 8:**
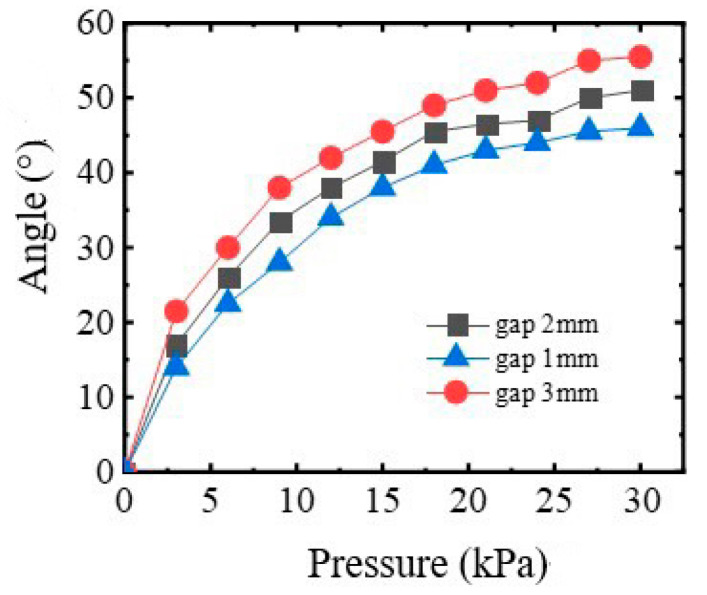
The effect of gap on the performance of rotating actuator for four fingers.

The influence of distance between air chambers on the performance angle variation is illustrated in Figure 8, and it can be found that when the air pressure exceeds 27 kPa, the angle variation is basically unchanged. Considering the rotating angle cannot exceed 45° and the initial size of SRGs, the best choice for the distance between air chambers is 1 mm.

**Figure 9:**
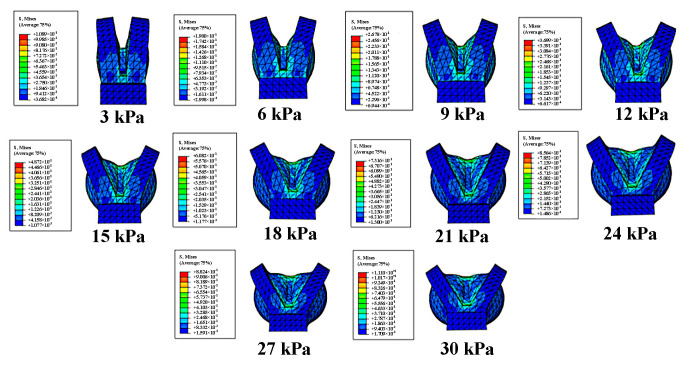
Simulation results of rotating angle.

**Figure 10:**
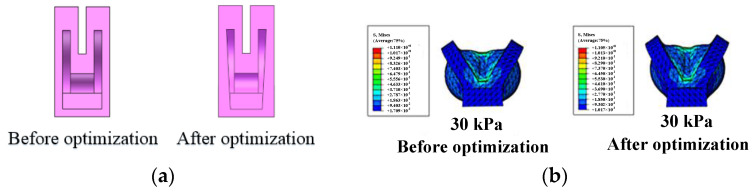
Optimization of the four-finger rotating. (**a**) Structural optimization and (**b**) optimization simulation comparison results.

The rotating angle under various pressures is shown in Figure 9; it is easy to see that with the increases of pressure applied, the rotating angle keeps increasing. Moreover, it can be seen that after air pressure exceeds 24 kPa, some of the elastic energy is consumed in the lateral deformation of sidewalls, which will not help to improve the rotating angle. To improve the angle output and reduce the energy loss, the optimized air chamber structure is shown in the Figure 10, and from the simulation result under 30 kPa, the expansion of the sidewall is slightly reduced.

**Figure 11:**
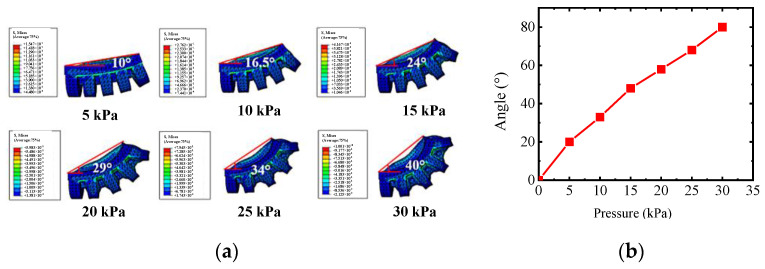
Simulation results of rotating actuator for thumb. (**a**) Simulation results of thumb actuator and (**b**) simulation curve of thumb.

Figure 11 illustrates the relationship between the rotating angle and pressure; in addition, the central angle is two times the rotating angle, so the central angle under various pressure is shown in Figure 11b. The angle output can meet the rehabilitation training.

**Figure 12:**
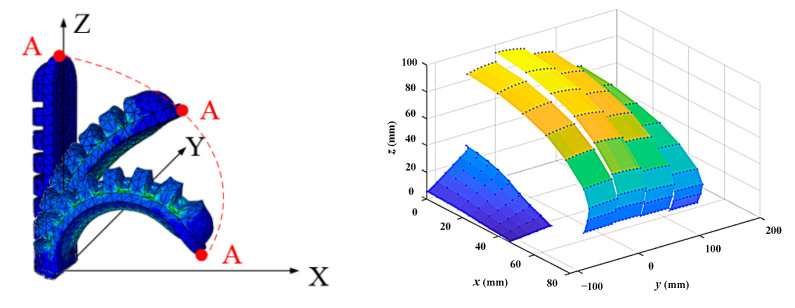
Workspace for SRGs.

The workspace is an important performance parameter for the evaluation of the robot. For SRGs, the overall working space is very complicated. To simplify this problem, we first analyzed the space of a single finger. Taking the index finger as an example, the coordinate system of the soft actuator must be established first to analyze the motion trajectory of the soft actuator. The coordinate system of the soft actuator is shown in Figure 12. The trajectory of point *A* for each actuator can be obtained through experiments, and the planar coordinate set (*X*, *Z*) of the end trajectory of the soft actuator is obtained. The motion trajectory of the soft rehabilitation glove is combining the bending angle of five soft actuators and the rotating angle between five fingers to form the working space. The thumb rotation needs to be studied separately; the rotating angles between the other four fingers are taken as the same. The range of the rotating angle for the four fingers was 0–30°, and the rotating angle for the thumb ranged from 0 to 80°. Using MATLAB to draw the trajectory of the endpoint for each actuator for each finger, the working space of the SRGs can be obtained.

## 4. Testing of Soft Bending Actuator for Glove

### 4.1. Experiments for Bending Actuators

**Figure 13:**
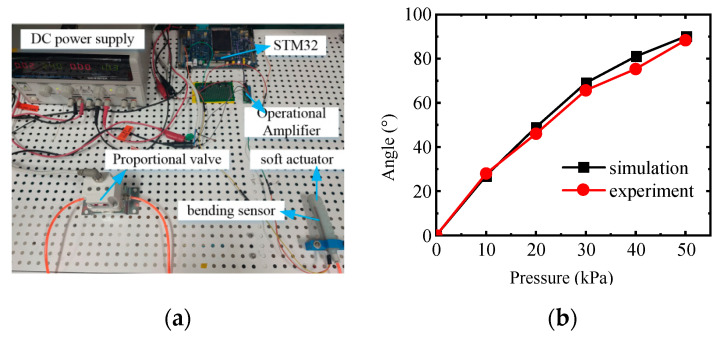
Bending angle test of bending actuator. (**a**) Bending Actuator Test Platform and (**b**) Bending Characteristics of Bending Actuators.

As shown in Figure 13, the experiment was carried out to test the bending angle under the air pressure of 0–50 kPa (interval of 10 kPa). The voltage value of the flexible bending sensor was acquired and converted into the bending angle of the actuator through a calibration experiment [27]. As can be seen from Figure 13b, the bending angle gradually increases with the increase in air pressure and agrees well with the simulation results.

**Figure 14:**
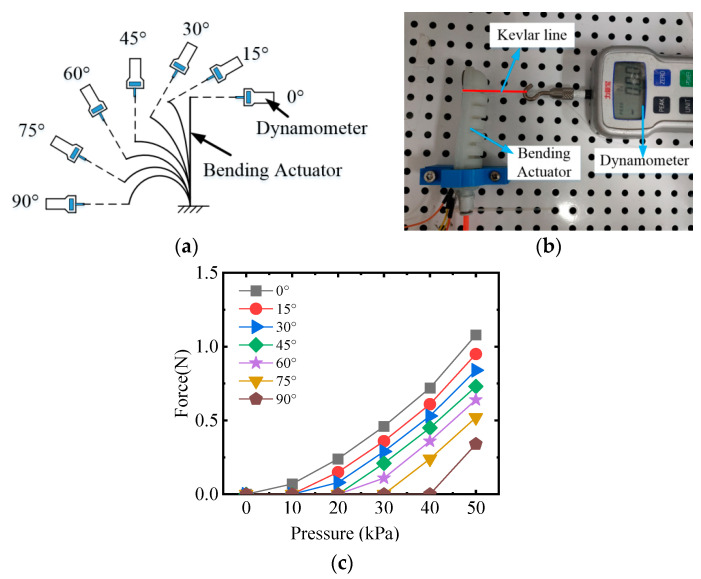
The force output test of the bending actuator. (**a**) Schematic diagram of the force test; (**b**) the force test device; and (**c**) bending angle of actuator.

This paper adopted a dynamometer (Kyoto, Japan, SHIMPO FGP-20) to measure the force output of the endpoint for the bending actuator; the schematic diagram of the measurement experimental platform is shown in Figure 14. When testing the force, the actuator was fixed on the platform and the dynamometer was connected to the endpoint of the actuator with Kevlar wire.

From the 0° initial position to the 90° position (with intervals of 15°), the force output was recorded sequentially under various applied pressures (10 kPa to 50 kPa). The measurement results show that the end force increases gradually as the air pressure increases. Under the same air pressure, the closer to the initial position, the greater the force. The reasons for these phenomena are easy to explain. In the working process of the pneumatic actuator, the work performed by compressed air is converted into the elastic strain energy and the force output. At positions closer to the 0° initial position, the elastic strain energy is smaller, thus the force output will be larger.

### 4.2. Experiments on Rotating Actuators

(1) Rotating angle and force output of rotating actuator for four fingers.

**Figure 15:**
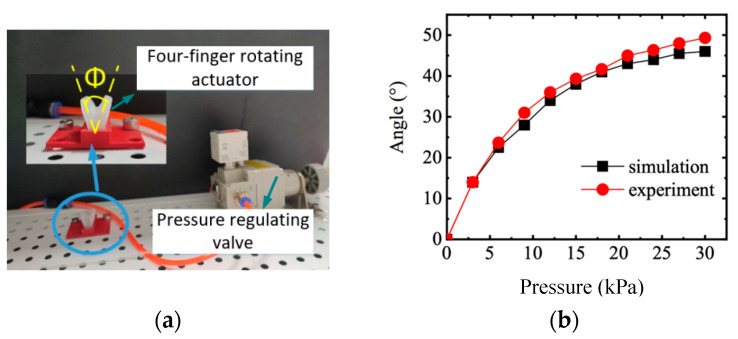
Angle test of rotating actuator. (**a**) Angle test. (**b**) Angle of rotating actuator.

The abduction and adduction motion of the four fingers is realized by the rotating of the actuator. As shown in Figure 15, adjusting the air pressure under 0–30 kPa with the valve, the rotating angle was tested. The experimental results agree well with the simulation analysis, though the experimental results are slightly larger than the simulation results.

**Figure 16:**
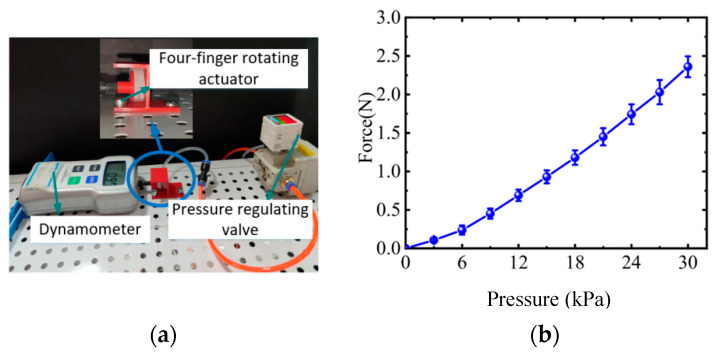
Mechanical characteristics test of rotating actuator for four fingers. (**a**) Force test of rotating actuator. (**b**) Force–pressure curve of rotating actuator.

As shown in Figure 16, a fixture was printed via 3D printer to clamp the rotating actuator on the table and the dynamometer was used to test the force output of the actuator under various pressures. It can be seen that with the increase of air pressure, the force also gradually increases. In the low pressure range of 0–6 kPa, the rate of the force of rotating actuator is smaller than that in the pressure range of 6–30 kPa. This may be caused by the nonlinearity of soft material.

(2) Rotating angle and force output of rotating actuator for thumb.

**Figure 17:**
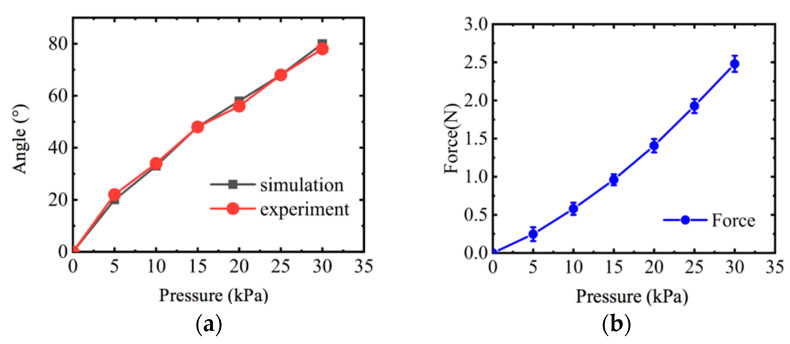
Angle and force test of actuator for thumb. (**a**) Thumb rotating angle results. (**b**) A curve of force versus pressure.

The experimental results are shown in Figure 17; it is easy to find that both the angle and force increase with the air pressure and the angle test data are consistent with the simulation results. The trends of the two curves are similar to the corresponding curves of the rotating actuator for four fingers.

## 5. Soft Rehabilitation Glove Control System

**Figure 18:**
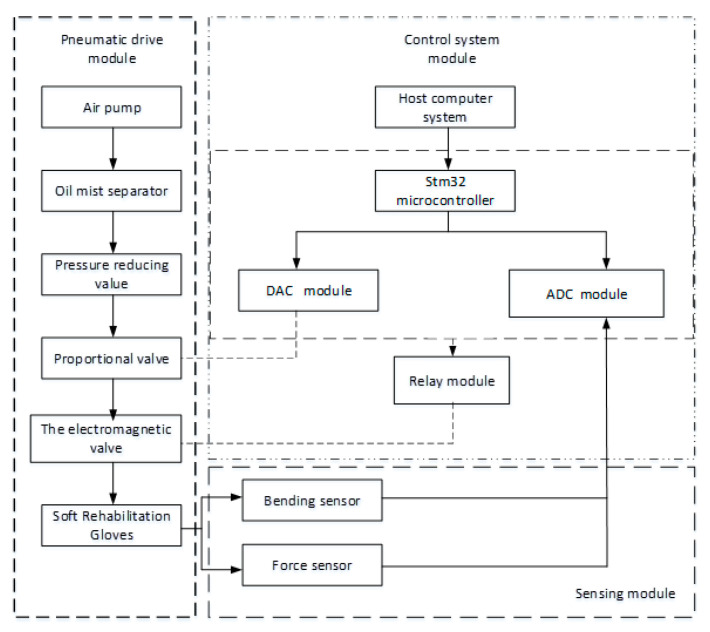
Schematic diagram of hardware system for SRGs.

To meet the working requirements of SRGs, the hardware system mainly consists of a pneumatic drive module, control system module, and sensor module, as shown in Figure 18. The pneumatic drive module is composed of an air pump (OUTSTANDING OTS-550), oil mist separator, solenoid valve (AIRTAC 3V2-08-NC), and proportional valve (SMC ITV1010-312BL). The control algorithm is implemented in a STM32. The working process of SRGs can be implemented by controlling the on–off of the solenoid valve and the voltage of the proportional valve to regulate the air pressure. The sensing module includes a flexible angle sensor and a force sensor, which is used to measure the bending angle and contact force of each finger.

**Figure 19:**
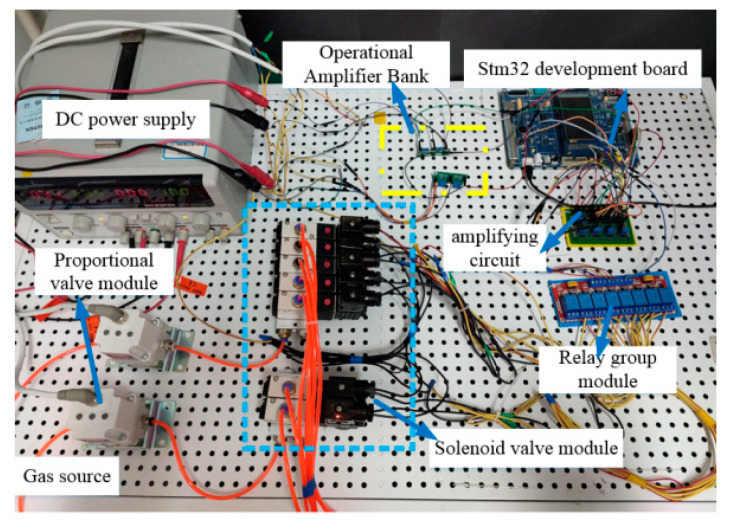
Physical map of the control platform.

According to the schematic diagram above, the experimental platform for SRGs is shown as Figure 19.

**Figure 20:**
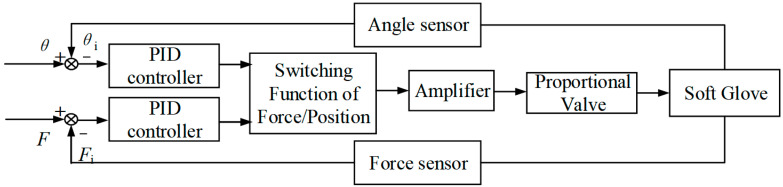
Control strategy for soft rehabilitation gloves.

Usually, position control is needed when the SRG is worn on a patient’s hand for rehabilitation training. However, force control should be executed when the SRG is used to assist grasping action. As shown in Figure 20, the switching function can realize the switching between force control and position control. Moreover, if SRGs are used to train the hand muscle at some desired angle position, the strategy of force control based on position control, also known as force/position hybrid control, will be employed.

PID controller is used here to improve control performance due to the nonlinearity and complex of the soft actuator. In order to reduce the calculation amount of the processor, the incremental PID is improved on the basis of the position PID, which is shown as follows

(4) $$\begin{array}{l} \Delta u(k)=u(k)-u(k-1)\\ =K_{p}\left[e(k)-e(k-1)\right]+K_{i}e(k)+K_{d}\left[e(k)-2e(k-1)+e(k-2)\right] \end{array}$$

where *e*(*k*), *e*(*k*−1), and *e*(*k*−2) is respectively the deviation value of the *k*th, (*k*−1)th, and (*k*−2)th sampling of the system for the angle *θ* and contact force *F*.

**Figure 21:**
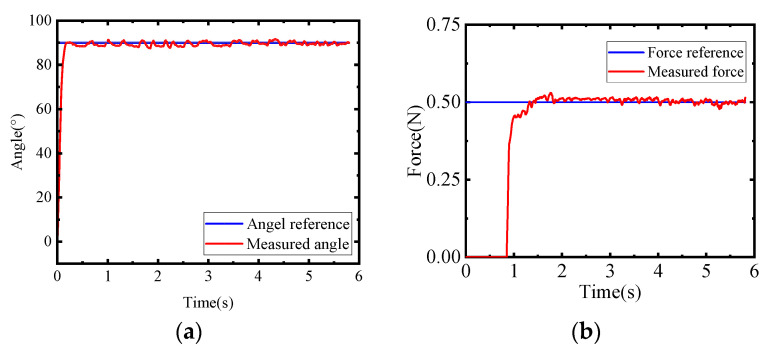
Position and force control experiment results. (**a**) Angle control experiment results (**b**) Force control experiment results.

The experimental data for angle position and contact force were collected using STM32, with a sampling frequency of 256 Hz. In this experiment, the angle reference and force could be chosen according to the demand of rehabilitation and assistive grasping within the motion range of SRGs. By automatically adjusting the PID parameters, the control signal of the voltage is output from STM32 to the proportional valve through the amplifier for controlling each soft actuator. As can be seen from Figure 21a, the finger can reach the angle position of 90° within 0.5 s with a deviation of ±2°. For example, the SRGs are used to grasp an object with 0.5 N on each actuator at an angle of 30°. Firstly, the PID position control is adopted to fulfill position control with adjusted PID parameters (*k*_p_ = 0.01, *k*_i_ = 0.067, *k*_d_ = 0.0001). When the soft actuator reaches the specified position and touches the object, it switches to the force algorithm control through the switching function, and the force control algorithm works. The PID parameters of the force algorithm are *k*_p_ = 0.06, *k*_i_ = 0.08, *k*_d_ = 0.1. As can be seen from Figure 21b, the desired force 0.5 N was reached within 1 s with a bias of 6%, and gradually became stable. In general, the soft actuator is difficult to control due to the material nonlinearity and large deformation of soft material. However, the SRGs can be effectively controlled with the PID algorithm.

## 6. Experiments of Soft Rehabilitation Gloves

**Figure 22:**
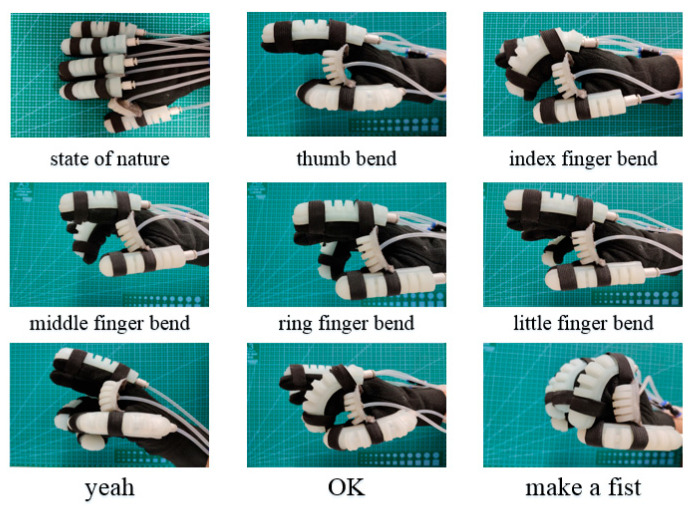
Experimental of gesture training for SRGs.

Figure 22 (Rotating joints work at 0–30 kPa air pressure and bending joints work at 0–50 kPa air pressure) shows some motion and gesture experimentation of soft rehabilitation gloves. Firstly, different instructions are sent to the host computer, and the soft rehabilitation gloves will execute the preset programs according to the instructions to achieve different postures. As illustrated in Figure 22, examples include the bending motion of every finger, the gesture “yeah”, and clenching a fist. The experimental results demonstrate that the soft rehabilitation gloves can realize various movements of the human hand and meet the requirements of rehabilitation training.

**Figure 23:**
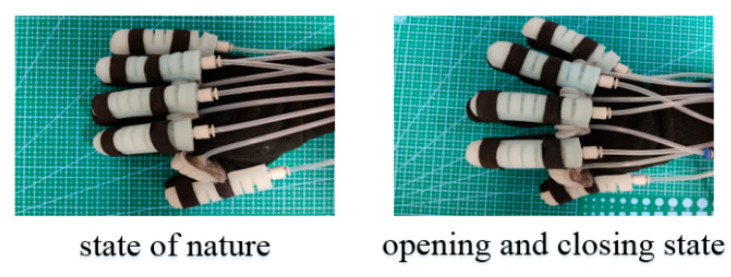
Abduction and adduction of SRGs.

In order to verify that the rehabilitation glove can realize the abduction and adduction function of the human hand, the opening and closing test of the soft rehabilitation glove was carried out. As shown in Figure 23 (rotating joints work at 0–30 kPa air pressure and bending joints work at 0–50 kPa air pressure), the corresponding action shows that the designed soft rehabilitation gloves can not only realize the bending rehabilitation training of the fingers, but also realize the abduction of the fingers, which further improves the rehabilitation training effect for the patients with finger hemiplegia.

**Figure 24:**
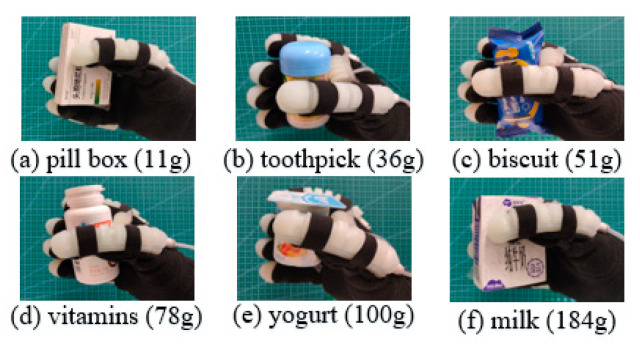
Grasping experiment.

To verify the assistance ability of SRGs, grasping experiments were performed; as shown in Figure 24, the soft rehabilitation gloves were worn on the hands of volunteers to grab common objects in life, such as pill boxes, vitamins, milk, etc., and the experiment demonstrated the SRGs could implement grasping action effortlessly. During the experiment, the volunteer did not experience uncomfortable feelings. This proves the SRGs could assisted the human hand to grasp objects, as well as accomplish the training motion within the normal range. In order to obtain better mechanical capacities, steel materials would be considered to enhance the holding and grasping force in the future work [28].

## 7. Conclusions

In this paper, a novel soft rehabilitation glove that has extension/flexion and abduction/adduction functions for every finger was presented. Firstly, the design requirements of the soft actuator were investigated and analyzed, and the structures of the bending actuator and the rotating actuator were designed based on the deformation difference principle of the strain layer and the restricted layer. We used 3D printing technology and a casting method to fabricate soft gloves with a flexible strain/force sensor embedded in. The influence of structure parameters on the mechanical behaviors upon pressurization was then studied using ABAQUS to improve the actuation performance. The workspace of the entire rehabilitation glove was drawn using MATLAB software (MathWorks, R2018a, Nedic, MA, USA).

Furthermore, the experiments on the output motions and forces of the bending actuator and rotating actuator were conducted and compared with the FEM simulation results. The hardware system of the soft rehabilitation gloves and the force/position PID control algorithm for SRGs were realized on the experimental platform. Finally, it was shown that the SRGs can successfully assist the human hand to accomplish various gestures and grasping motions.

This work mainly focused on structure design and fabrication, mechanical performance, and the control algorithm of soft rehabilitation gloves. Flexible strain sensors and force sensors were integrated into the SRGs to monitor the angle and force. Our job provides a basic platform for the hand rehabilitation and assistance grasping for patients with hand injuries. The limitation of the current research is that the rotating actuator occupies the room between each finger of the soft glove, which should be solved in the future. In addition, we will accomplish the optimal design of SRGs and exploit the force/position hybrid control algorithm in depth.

## Figures and Tables

**Table 1:** Four-finger bending actuator structure scheme.

Structure Parameter/mm	Outer Diameter of the Air Chamber b_1_	Interval c_1_	Inner Diameter of the Air Chamber t_1_	Thickness of Restricted Layer h_1_	Length L_1_
Plan a	3	1	1	1	7
Plan b	3	1	2	1	7
Plan c	3	1	0.5	1	7
Plan d	3	2	1	1	8
Plan e	3	3	1	1	9
Plan f	3	1	1	0.5	7
Plan g	3	1	1	2	7

**Table 2:** Structure Scheme of Thumb rotating Actuator.

Structure Parameter/mm	Outer Diameter of the Air Chamber b_2_	Interval c_2_	Inner Diameter of the Air Chamber t_2_	Thickness of Restricted Layer h_2_	Length L_2_
Plan	5	2	2	2	33

## Data Availability

All test data mentioned in this paper will be made available upon request to the corresponding author’s email with appropriate justification.
